# Supplementary material for: The causal role of circulating inflammatory markers in osteoporosis: a bidirectional Mendelian randomized study
Source: Front Immunol. 2024 Jul 18;15:1412298. doi: 10.3389/fimmu.2024.1412298 (PMC11291241; doi:10.3389/fimmu.2024.1412298)
Supplement: Supplementary file 1 [file DataSheet_1.zip › supplementary material/CIM-OSTEOPOROSIS /GCST90274812.csv_forest.pdf]

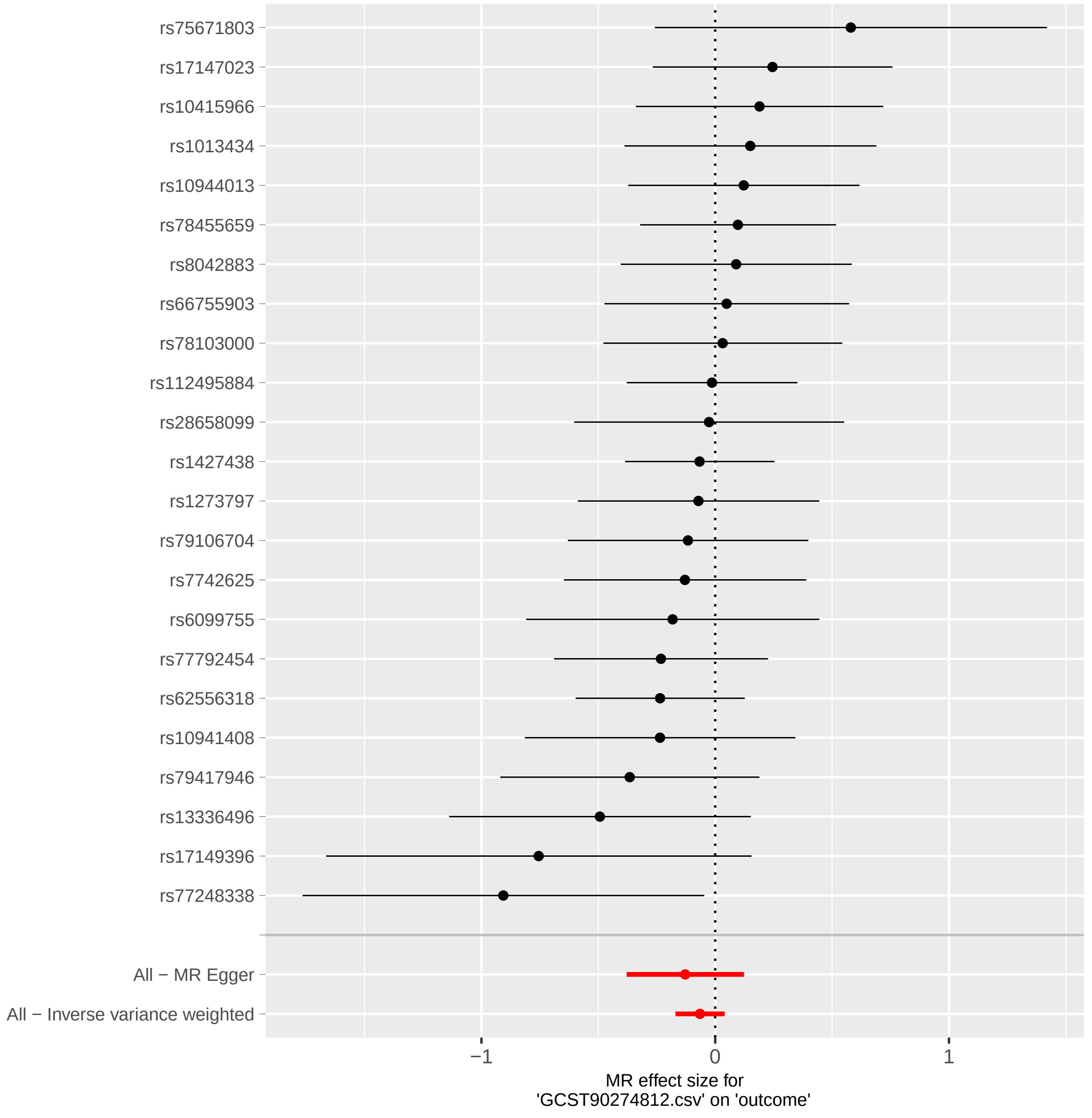
rs75671803
rs17147023
rs10415966
rs1013434
rs10944013
rs78455659
rs8042883
rs66755903
rs78103000
rs112495884
rs28658099
rs1427438
rs1273797
rs79106704
rs7742625
rs6099755
rs77792454
rs62556318
rs10941408
rs79417946
rs13336496
rs17149396
rs77248338
All − MR Egger
All − Inverse variance weighted
−1
0
1
MR effect size for
'GCST90274812.csv' on 'outcome'
